# Disturbed Eating Behaviors in Youth with Type 1 Diabetes: An Exploratory Study about Challenges in Diagnosis

**DOI:** 10.3390/diagnostics10121044

**Published:** 2020-12-03

**Authors:** Valeria Calcaterra, Chiara Mazzoni, Donatella Ballardini, Elena Tomba, Gian Vincenzo Zuccotti, Chiara Mameli, Rachele De Giuseppe, Hellas Cena

**Affiliations:** 1 Pediatric and Adolescent Unit, Department of Internal Medicine, University of Pavia, 27100 Pavia, Italy; valeria.calcaterra@unipv.it; 2 Department of Pediatrics, “Vittore Buzzi” Children’s Hospital, 20157 Milano, Italy; gianvincenzo.zuccotti@unimi.it (G.V.Z.); chiara.mameli@unimi.it (C.M.); 3 Eating Disorder Clinic “Centro Gruber”, 40125 Bologna, Italy; chiara.mazzoni88@gmail.com (C.M.); donatella.ballardini@yahoo.com (D.B.); 4 Department of Psychology, University of Bologna, 40125 Bologna, Italy; elena.tomba@unibo.it; 5 Department of Biomedical and Clinical Science “L. Sacco”, University of Milano, 20157 Milano, Italy; 6 Laboratory of Dietetics and Clinical Nutrition, Department of Public Health, Experimental and Forensic Medicine, University of Pavia, 27100 Pavia, Italy; rachele.degiuseppe@unipv.it; 7 Clinical Nutrition and Dietetics Service, Unit of Internal Medicine and Endocrinology, ICS Maugeri IRCCS, 27100 Pavia, Italy

**Keywords:** type 1 diabetes, disordered eating behaviors, eating disorders, youth, adolescent, subscales

## Abstract

Background: Disordered eating behaviors (DEBs), including diagnosable eating disorders, are quite common and can interfere with optimal type 1 diabetes (T1DM) management. We explored DEBs prevalence in youth with T1DM, proposing news diagnostic subscales, to represent the clinical dimensions associated with feeding and eating disorders (ED); Methods: additionally to SCOFF questionnaire and Diabetes Eating Problem Survey–Revised (DEPS-R), four subscales combined from the original DEPS-R questionnaire were administered to 40 youths with T1DM (15.0 ± 2.6); Results: females showed higher scores than males in DEPS-R original factor 2 (“preoccupations with thinness/weight”, *p* = 0.024) and in DEPS-R proposed “restriction” factor (*p* = 0.009). SCOFF scores was correlated with original DEPS-R factors 1 (“maladaptive eating habits”) and 2 (*p* < 0.001) and with the newly proposed DEPS-R factors: restriction, disinhibition, compensatory behaviors, diabetes management (all *p* < 0.02). Diabetes management was the only factor related to glycated hemoglobin level (*p* = 0.006). Patients with high DEPS-R score (≥20) scored higher than patients with low (<20) DEPS-R score in DEPS-R original factors 1 (*p* < 0.001) and 2 (*p* = 0.002) as well as in the proposed factors including restriction, disinhibition, diabetes management (all *p* < 0.02); Conclusions: the complicated nature of DEBs calls for the development target specific questionnaires to be used as screening tools to detect cases of DEBs and exclude non cases. Early recognition of DEBs in adolescents with T1DM is essential for effective prevention and successful treatment.

## 1. Introduction

Type 1 diabetes mellitus (T1DM) is a common chronic disease in children and young people, caused by autoimmune destruction of beta-pancreatic cells and characterized by chronic hyperglycemia [1,2]. Self-managing T1DM requires frequent checking of blood glucose levels, carbohydrate (CHO) counting to estimate CHO content per meal, effective responses to exercise, and self-injecting insulin in adjusted doses [1,2,3]. These burdens are considered predisposing factors for mental disorders, including eating disorders (ED), with a 2–3 fold increase in prevalence compared to individuals without T1DM [4,5,6,7].

According to Diagnostic and Statistical Manual of Mental Disorders-5 (DSM-5; APA, 2013), Feeding and Eating Disorders (FED) are specifically identified by defined criteria for which a degree of severity has been established, such as Anorexia Nervosa (AN), Bulimia Nervosa (BN), Binge Eating Disorder (BED), Other Specified Feeding and Eating Disorder (OSFED), Pica, Rumination, Avoidant/Restrictive Food Intake Disorder (ARFID), and also Unspecified Feeding or Eating Disorder (UFED).

These categories include a wide spectrum of eating behavior disturbances that must be closely evaluated such as selective eating, recurrent restrictive diets, binge eating, recurrent use of inappropriate compensatory behaviors to prevent weight gain, including fasting, excessive exercise, self-induced vomiting, misuse of laxative or diuretic, drinking excessive amounts of water or non-caloric beverages, mints, and gum [8,9,10,11,12,13,14].

Disordered eating behaviors (DEB), including diagnosable eating disorders, in the context of diabetes, are quite common and can directly interfere with optimal diabetes management [4,5,6,7].

DEB is more common in females with T1DM and is frequently associated with insulin omission for weight control and consequent higher rates of diabetes-related medical complications and tripled risk of mortality [4,5,6,7,15].

However, DEBs remain underdiagnosed, exposing teens to higher rates of disease complications. It is therefore important to pay attention to clinical warning signs that raise suspicion of disturbed eating and introduce the use of screening tools in clinical practice that could help practitioners to refer these patients early to an expert in clinical nutrition and eating disorders [16,17].

Usually, FEDs and DEBs are assessed by means of screening questionnaires for EDs, not specific for individuals with T1DM. Mostly questionnaires do not include T1DM specific items, such as insulin omission [6,8,18,19].

Furthermore, questionnaires tend to consider certain eating habits and behaviors that are integral to diabetic care, including attention to diet, foods selection and avoidance, and eating when not hungry, as expressions of disordered eating [8,18,19]. The Diabetes Eating Problem Survey—Revised (DEPS-R) was the first screening tool for disordered eating specifically designed for diabetes and validated in several different populations [12,13,14,18,20,21]. However, as reported by Ryman [22], a pathological DEPS-R score ≥20 is not specific for FED diagnosis according to DSM-5, since other variables, including psychosocial characteristics and diabetes conflict, should be considered in association with an abnormal DEPS-R screen.

Moreover, in the field of clinical medicine and psychiatry the merely use of the psychometric model to obtain screening tools by means of statistical procedures, such as factor analysis, has been challenged [23,24,25,26] since not always able to detect real clinical phenomena. In psychometrics, the same properties that provide high score scale for homogeneity may obscure ability to detect change, and redundant scale items may increase homogeneity, but decrease sensitivity [27] with the consequent effect of drafting screening tools not accurate in differentiating appropriately the clinical variables.

The clinimetric perspective [28] offers an integrative approach to psychometrics in the measurement of clinical variables in medicine and psychiatry proposing an integration of the clinical reasoning for the development of appropriate clinical screening tools. Following the clinimetric prospective this exploratory study aimed at proposing an alternative method to collect data and estimate DEBs from a clinical perspective by means of DEPS-R questionnaire, in a specific population.

In particular, the definite items and subscales of DEPS-R detecting symptoms of feeding and eating disorders have been better identified and redistributed according to DSM-5 criteria and compared to DEBS-R original subscales, as derived from factor analysis, in order to better identify disturbances of eating behaviors in youths with T1DM, even in an exploratory design.

In this exploratory study, we proposed four subscales composed from the original DEPS-R subscales to early feeding and eating disorders detection in youth with T1DM.

## 2. Methods

### 2.1. Patients

In this study 40 youth with T1DM (17 females and 23 males), aged between 11–18 years (mean age 15.0 ± 2.6 years) were recruited and followed up in the outpatient Pediatrics Diabetology Unit at IRCCS Policlinico San Matteo, Pavia, Italy, between June 2016 and June 2017. The sample characteristics are resumed in Table 1. In all patients, diagnosis was made at least 12 months before their recruitment, and an anamnestic diagnosis of FED was considered an exclusion criterion. All patients were treated with a basal-bolus insulin regimen by means of multiple daily injections or continuous subcutaneous insulin infusion (insulin pump).

The study protocol (EC AOU Policlinico V. Emanuele, CA, n.203/2017/PO) was run in accordance with the Helsinki Declaration of 1975, as revised in 2008. All participants or their responsible guardians were asked for and gave their written consent after being informed about the nature of the study.

### 2.2. Tools

The study included both a non-standardized interview to collect socio-demographic, anthropometric data, and medical history, besides two standardized self-report psychometric questionnaires:

(1) the SCOFF (Sick, Control, One stone, Fat, Food) questionnaire [29,30], a 5-items psychometric self-report screening questionnaire for detecting EDs. Items in the SCOFF require “yes” (=1) or “no” (=0) answers and total score is sum of the individual item scores. Total score ranges from 0–5 with a cut-off of >2 indicating a possible ED and the need of further evaluation. Although reliability is fairly low in the Italian version (Cronbach’s alpha of 0.64) due to the low number of items, the SCOFF questionnaire has shown good discriminative power between ED and non-ED individuals [30];

(2) the Diabetes Eating Problem Survey—Revised (DEPS-R) [19], a 16-items diabetes-specific psychometric self-report screening tool for disordered eating behaviors derived from the Diabetes Eating Problem Survey (DEPS) [31]. Items are scored on a 6-point Likert scale, with higher scores indicating greater DEBs in patients with diabetes. DEPS-R cut-off was empirically set at 20 to screen for patients with a level of disordered eating that might warrant further investigation. From previous validation studies three factors emerged for this questionnaire: 1. Maladaptive eating habits, 2. Preoccupation with thinness or weight, and 3. concept of maintaining high blood glucose values to lose weight [12,13,32]. The Italian version of the DEPS-R also demonstrated a good internal consistency with a Cronbach’s alpha of 0.83 [32].

(3) In the current study DEPS-R items detecting feeding and eating disorders symptoms have been redistributed according to the DSM-5 FED criteria. Four subscales have been then composed from the original DEPS-R subscales [32], in order to provide a more accurate representation of the clinical dimensions associated with feeding and eating disorders in patients with diabetes. The new DEPS-R four subscales proposed, which are hypothesized to be associated with a positive DEPS-R screening optimized diagnosis, are: 1. Restriction and body dissatisfaction, 2. Disinhibition, 3. Compensatory behaviors, and 4. Diabetes management (see Table 2).

### 2.3. Statistical Analyses

T-tests for independent samples and correlation analyses were run in the whole sample (*n* = 40). T-tests for independent samples were used to compare scores in the DEPS-R original and proposed factors between males (*n* = 23) and females (*n* = 17). Pearson’s bivariate correlation analyses were also conducted to examine correlations between DEPS-R original and proposed factors and SCOFF scores, BMI, and levels of glycated hemoglobin (HbA1c) in the whole sample.

Participants were then divided into two groups according to the DEPS-R total score using a cut-off of 20. T-tests for independent samples were used to compare scores in the original and proposed DEPS-R scores between patients with a DEPS-R total score (≥20) and patients with a low DEPS-R total score (<20). Pearson’s bivariate correlation analyses were conducted to examine correlations between DEPS-R original and proposed factors and SCOFF scores, BMI, and levels of glycated hemoglobin (HbA1c) in the subgroup of patients reporting a DEPS-R total score ≥ 20 (*n* = 8). All analyses were performed using the Statistical Package for the Social Sciences (SPSS) software version 14.0 (SPSS Inc., Chicago, IL, USA). Statistical significance was set at a *p*-level of 0.05.

## 3. Results

### 3.1. Comparison between DEPS-R Original Subscales vs. DEPS-R Newly Proposed One in the Whole Sample

T-tests for independent samples (Table 3) in our sample showed that females scored higher than males in DEPS-R original factor 2 (“preoccupations with thinness or weight”) (*t*_(24.62)_ = −2.408, *p* = 0.024) and in newly proposed DEPS-R “restriction” factor (*t*_(38)_ = −2.751, *p* = 0.009). No significant differences were found between gender in DEPS-R original factors 1 and 3 (“maladaptive eating habits” and “concept of maintaining high blood glucose values to lose weight”) as well as in the newly proposed DEPS-R “disinhibition”, “compensatory behaviors”, and “diabetes management” factors.

### 3.2. Correlation between SCOFF, HbA1c, DEPS-R Original Subscales vs. DEPS-R Newly Proposed One in the Whole Sample

Pearson’s bivariate correlation analyses run in the total sample (Table 4) showed significant positive moderate correlations between SCOFF scores and original DEPS-R factors 1 (“maladaptive eating habits”) (*r* = 0.463, *p* < 0.001) and 2 (“preoccupations with thinness and weight”) (*r* = 0.516, *p* < 0.001). Scores in the SCOFF questionnaire also correlated positively, moderately and significantly with the four newly proposed DEPS-R factors: restriction (*r* = 0.380, *p* = 0.016), disinhibition (*r* = 0.405, *p* = 0.010), compensatory behaviors (*r* = 0.467, *p* = 0.002), and diabetes management (*r* = 0.405, *p* = 0.010). The newly proposed DEPS-R diabetes management factor was the only one showing a significant correlation with the levels of glycated hemoglobin (HbA1c) (*r* = 0.434, *p* = 0.006).

### 3.3. Comparison between DEPS-R Original Subscales and DEPS-R Proposed Subscales in Patients at Risk of EDs

T-tests for independent samples run into two groups according to the DEPS-R total score using a cut-off of 20 highlighted that patients with high (≥20) DEPS-R score had significantly higher scores than patients with low (<20) DEPS-R score (Table 5), both in DEPS-R original factors 1 (“maladaptive eating habits”) (*t*_(38)_ = −5.119, *p* < 0.001) and 2 (“preoccupations with thinness or weight”) (*t*_(8.05)_ = −4.523, *p* = 0.002). They also reported higher scores in the proposed DEPS-R restriction (*t*_(7.67)_ = −3.185, *p* = 0.014), disinhibition (*t*_(38)_ = −4.910, *p* < 0.001), and diabetes management (*t*_(38)_ = −3.903, *p* < 0.001) factors.

When selecting only patients (*n* = 8) with high (>20) DEPS-R score, correlation analyses did not show any correlation between original or newly proposed DEPS-R factors with SCOFF scores, BMI, diabetes duration, nor levels of glycated hemoglobin (Table 6).

## 4. Discussion

T1D is the most common chronic endocrine disease in youth. Living with diabetes type 1 is a major challenge including multiple significant practical tasks. Self-managing T1D entails frequent monitoring of blood glucose, carbohydrate (CHO) counting in meals, physical activity effect, and self-injecting insulin [33]. These major changes and role transitions are predisposing factors for mental disorders [33].

In particular, youth with T1D have been found to be at-risk for DEBs development, as suggested be the prevalence data indicating up to 40% sub-clinical DEBs in T1D [6]. Female gender, teen age, especially between 13 and 14 years for girls and above 16 years for boys, body weight [16] and body dissatisfaction, constant fixation with food and presence of other psychiatric disorders, such as depression, anxiety, are key risk factors for ED development [9]. Furthermore, in individuals affected by T1DM, ED are associated with poor diabetes control, clinical complications and increased mortality rates as compared to age-matched population [9,15]. On the other hand emphasis on food portion size, CHO counting, weight management may contribute to develop rigid thinking about food, dieting, weight, and body shape, besides higher diabetes-related family conflicts, frequent in adolescence, increasing risk for both ED and for poor glycemic control [34,35].

Even thought, identification of the most appropriate tools to screen for DEBs in T1D plays a crucial role in effective prevention and consequent morbidity and mortality decrease as well as treatment outcomes, appropriate tools for DEB assessment in T1D are still lacking.

In this study, a modified SCOFF version for T1D has been considered as a first step screening tool for adolescents useful in clinical settings, suggesting that SCOFF score ≥2 warrants further assessment for disordered eating. Up until now, DEPS-R has been the most widely translated and validated tool for DEB assessment in adolescents with T1D, with high internal reliability, concurrent, criterion and convergent validity [7]. DEPS-R is a 16-item questionnaire for general and diabetes-specific DEB assessment, including weight loss, food restriction, insulin measure and vomiting. The Italian version of this tool has shown significant correlations between total score and BMI, HbA1c both in males and females, aged 15–55 years [32].

Our results confirmed the correlation between DEPS-R score and HbA1c, in particular showed that sub-scaling according to DSM-5 criteria could be more useful in outlining clinical characteristics, depicting eating behavior disorders among those identified as potentially at risk by the original DEPS. Noteworthy, from our result we may also hypothesize that eliminatory conduct is not likely to distinguish individuals at risk or not.

A significant positive moderate correlation has been observed between SCOFF score and original DEPS-R factors 1 and 2; besides SCOFF score also correlated with all four proposed DEPS-R factors, including restriction, disinhibition, compensatory behaviors, and diabetes management. Moreover, when selecting patients with DEPS-R score ≥ 20, those correlations disappeared. Therefore it could be assumed that DEPS may be useful for DEB screening, nevertheless in patients with DEPS-R score ≥ 20, the use of proposed subscales based on DSM-5 criteria could additionally help in defining a proper clinical classification. This study has some limitations, including the small sample size, and lack of details on the diabetes-related impact upon families and caregivers. Therefore, further studies are necessary to confirm our results. Despite such limitations, studies like this one, focused on the proposal of new subscales, associated with positive DEPS-R screening, may be very important for early diagnosis and personalized treatment optimization. This is in line with the clinimetric perspective introduced by Feinstain [28] which offers the conceptual and methodological path for a substantial revision of assessment parameters in clinical medicine and psychiatry. Such switch may promote significant clinical and research advantages. By a clinical viewpoint, it may allow more clinical flexibility and may help professionals be more in tune with the clinical reasoning, both in terms of diagnostic assessment and evaluation of concurrent comorbid conditions. In terms of seeking good solutions for further progress, our results pave the path for developing target specific assessment tools more suitable and appropriate for clinical research purposes. Rigid adherence to the psychometric model may only limit such progress [24,25,26].

## 5. Conclusions

In this study, we explored DEBs prevalence in youth with T1DM, proposing new diagnostic subscales to represent the clinical dimensions associated with feeding and eating disorders in this clinical target.

We administered four subscales combined from the original DEPS-R questionnaire, besides SCOFF and DEPS-R questionnaires, to 40 youths with T1DM. After examining the results, our statistical analysis concluded that although DEPS-R may be useful for DEB screening, the proposed subscales based on DSM-5 criteria in patients with DEPS-R score ≥ 20 could additionally help in defining a proper clinical classification.

Although previous findings indicated high prevalence of DEB and EDs among teens with T1DM and strong association with long-term medical complications, our study showed the need to develop instruments able to detect the specific dysfunctional pattern of EDs, that may help early recognition in youth affected by TD1M.

Future researchers should consider investigating the impact in these patients of other diabetes-specific control behaviors on eating disorders.

Regardless, our results point to the need for medical practitioners and pediatricians to consider DEBs early detection during first line evaluation in order to refer these patients as soon as possible to experts in clinical nutrition and mental health disorders for positive outcomes for T1DM adolescents.

## Figures and Tables

**Table 1:** Characteristics of the enrolled patients.

Characteristics	Value
Number of patients (*n*)	40
Age at evaluation (years) ^	15.0 ± 2.6
Age at the pathology onset (years) ^	4.7 ± 2.2
Sex (M/F)	23/17
Weight (kg) ^	62.7 ± 12.4
Height (m) ^	1.6 ± 9.8
BMI (kg/m^2^) ^	22.8 ± 3.1
BMI z-score ^	0.6 ± 1.0
HbA1c (%) ^	8.3 ±1.6
Insulin regimen -multiple daily injections (%) -insulin pump (%)	36/40 (90) 4/40 (10)
Normal-weight patients * (%)	37/40 (92.5)

^ data are expressed as mean ± standard deviation. * Body mass index (BMI) < 75th percentile for age and sex.

**Table 2:** The original Diabetes Eating Problem Survey–Revised (DEPS-R) factors and related items and the new proposed factors and redistribution of the items.

**Original Factors**	**DEPS-R Items**
1. Maladaptive eating habits	Item 2 (I skip meals and/or snacks) Item 3 (Other people have told me that my eating is out of control) Item 4 (When I overeat, I don’t take enough insulin to cover the food) Item 5 (I eat more when I am alone than when I am with others) Item 7 (I avoid checking my blood sugar when I feel like it is out of range) Item 12 (Other people tell me to take better care of my diabetes) Item 13 (After I overeat, I skip my insulin dose) Item 14 (I feel that my eating is out of control) Item 15 (I alternate between eating very little and eating huge amounts)
2. Preoccupations with thinness or weight	Item 1 (Losing weight is an important goal to me) Item 6 (I feel that it’s difficult to lose weight and control my diabetes at the same time) Item 11 (I feel fat when I take all of my insulin) Item 16 (I would rather be thin than to have good control of my diabetes)
3. Concept of maintaining high blood glucose values to lose weight	Item 8 (I make myself vomit) Item 9 (I try to keep my blood sugar high so that I will lose weight) Item 10 (I try to eat to the point of spilling ketones in my urine)
**Proposed Factors**	**DEPS-R Items**
1. Restriction and body dissatisfaction	Item 1 (Losing weight is an important goal to me) Item 2 (I skip meals and/or snacks) Item 11 (I feel fat when I take all of my insulin) Item 16 (I would rather be thin than to have good control of my diabetes)
2. Disinhibition	Item 3 (Other people have told me that my eating is out of control) Item 5 (I eat more when I am alone than when I am with others) Item 14 (I feel that my eating is out of control) Item 15 (I alternate between eating very little and eating huge amounts)
3. Compensatory behaviors	Item 4 (When I overeat, I don’t take enough insulin to cover the food) Item 8 (I make myself vomit) Item 9 (I try to keep my blood sugar high so that I will lose weight) Item 13 (After I overeat, I skip my insulin dose)
4. Diabetes management	Item 6 (I feel that it’s difficult to lose weight and control my diabetes at the same time) Item 7 (I avoid checking my blood sugar when I feel like it is out of range) Item 10 (I try to eat to the point of spilling ketones in my urine) Item 12 (Other people tell me to take better care of my diabetes)

**Table 3:** *T*-tests for independent samples comparing males and females in DEPS-R original and newly proposed factors.

DEPS-R Factors	Gender	*n*	M ± SD	*t*_(df)_	*p*
ORIGINAL FACTOR 1 “Maladaptive eating habits”	Male Female	23 17	6.57 ± 4.1 39.71 ± 6.51	−1.747_(25.32)_ *	0.093
ORIGINAL FACTOR 2 “Preoccupations with thinness or weight”	Male Female	23 17	2.74 ± 2.85 5.82 ± 4.68	−2.408_(24.62)_ *	0.024
ORIGINAL FACTOR 3 “Concept of maintaining high blood glucose values to lose weight”	Male Female	23 17	0.26 ± 1.05 0.24 ± 0.75	0.085_(38)_	0.933
PROPOSED FACTOR 1 “Restriction and body dissatisfaction”	Male Female	23 17	2.61 ± 2.02 5.24 ± 3.95	−2.751_(38)_	0.009
PROPOSED FACTOR 2 “Disinhibition”	Male Female	23 17	3.43 ± 2.73 5.47 ± 3.96	−1.929_(38)_	0.061
PROPOSED FACTOR 3 “Compensatory behaviors”	Male Female	23 17	1.17 ± 0.887 1.47 ± 1.63	−0.682_(23.01)_ *	0.502
PROPOSED FACTOR 4 “Diabetes Management”	Male Female	23 17	3.91 ± 2.88 3.47 ± 3.71	0.425_(38)_	0.673

Abbreviations: DEPS-R: Diabetes Eating Problem Survey-Revised (Markowitz et al., 2010); M: Mean; *n*: number of participants; SD: Standard Deviation; *t*: Student’s t. * Levene’s test for equality of variances was found significant.

**Table 4:** Pearson’s bivariate correlation analyses between original and proposed DEPS-R factors and SCOFF scores, diabetes duration, BMI, and levels of H1bA1c in the total sample (*n* = 40).

DEPS-R Factors	SCOFF	Diabetes Duration (years)	BMI	H1bA1c
ORIGINAL FACTOR 1 “Maladaptive eating habits”	0.463 ^+^	0.248	0.038	0.283
ORIGINAL FACTOR 2 “Preoccupations with thinness or weight”	0.516 ^+^	−0.150	0.199	0.102
ORIGINAL FACTOR 3 “Concept of maintaining high blood glucose values to lose weight”	0.077	−0.235	0.122	0.088
PROPOSED FACTOR 1 “Restriction and body dissatisfaction”	0.380 *	−0.022	0.293	0.162
PROPOSED FACTOR 2 “Disinhibition”	0.405 **	0.065	−0.127	0.262
PROPOSED FACTOR 3 “Compensatory behaviors”	0.467 **	0.296	0.227	0.030
PROPOSED FACTOR 4 “Diabetes Management”	0.405 **	0.077	−0.013	0.434 **

Abbreviations: BMI: Body Mass Index; DEPS-R: Diabetes Eating Problem Survey-Revised (Markowitz et al., 2010); HbA1c: glycated hemoglobin; SCOFF: SCOFF questionnaire scores (Morgan, Reid, and Lacey, 2000). * *p* < 0.05; ** *p* < 0.01, ^+^ *p* < 0.001.

**Table 5:** T-tests for independent samples comparing patients with low (<20) and high (>20) DEPS-R scores in DEPS-R original and newly proposed factors.

DEPS-R Factors	DEPS-R Score	*n*	M ± SD	*t*_(df)_	*p*
ORIGINAL FACTOR 1 “Maladaptive eating habits”	Low (<20) High (≥20)	32 8	6.19 ± 3.86 14.75 ± 5.57	−5.119_(38)_	<0.001
ORIGINAL FACTOR 2 “Preoccupations with thinness or weight”	Low (<20) High (≥20)	32 8	2.63 ± 2.32 9.75 ± 4.30	−4.523_(8.05)_ *	0.002
ORIGINAL FACTOR 3 “Concept of maintaining high blood glucose values to lose weight”	Low (<20) High (≥20)	32 8	0.06 ± 0.246 1.00 ± 1.93	−1.37_(7.06)_ *	0.212
PROPOSED FACTOR 1 “Restriction and body dissatisfaction”	Low (<20) High (≥20)	32 8	2.72 ± 1.89 7.75 ± 4.37	−3.185_(7.67)_ *	0.014
PROPOSED FACTOR 2 “Disinhibition”	Low (<20) High (≥20)	32 8	3.25 ± 2.60 8.50 ± 3.12	−4.910_(38)_	<0.001
PROPOSED FACTOR 3 “Compensatory behaviors”	Low (<20) High (≥20)	32 8	1.03 ± 0.86 2.38 ± 1.92	−1.929_(7.72)_ *	0.091
PROPOSED FACTOR 4 “Diabetes Management”	Low (<20) High (≥20)	32 8	2.88 ± 2.50 7.13 ± 3.68	−3.903_(38)_	<0.001

Abbreviations: DEPS-R: Diabetes Eating Problem Survey-Revised (Markowitz et al., 2010); M: Mean; *n*: number of participants; SD: Standard Deviation; *t*: Student’s t. * Levene’s test for equality of variances was found significant.

**Table 6:** Pearson’s bivariate correlation analyses between original and newly proposed DEPS-R factors and SCOFF scores, diabetes duration, BMI, and levels of H1bA1c in patients with a high DEPS-R score (>20) (*n* = 8).

DEPS-R Factors	SCOFF	Diabetes Duration (years)	BMI	H1bA1c
ORIGINAL FACTOR 1 “Maladaptive eating habits”	0.136	−0.140	0.405	0.180
ORIGINAL FACTOR 2 “Preoccupations with thinness or weight”	−0.080	−0.513	0.435	0.415
ORIGINAL FACTOR 3 “Concept of maintaining high blood glucose values to lose weight”	−0.358	−0.481	−0.124	0.199
PROPOSED FACTOR 1 Restriction and body dissatisfaction	−0.300	−0.093	0.665	0.541
PROPOSED FACTOR 2 Disinhibition	0.000	−0.485	0.382	0.175
PROPOSED FACTOR 3 Compensatory behaviors	0.664	0.148	0.403	0.042
PROPOSED FACTOR 4 Diabetes Management	−0.009	−0.659	−0.006	−0.036

Abbreviations: BMI: Body Mass Index; DEPS-R: Diabetes Eating Problem Survey-Revised (Markowitz et al., 2010); HbA1c: glycated hemoglobin; SCOFF: SCOFF questionnaire scores (Morgan, Reid, and Lacey, 2000).
